# Isorhamnetin Protects against Doxorubicin-Induced Cardiotoxicity In Vivo and In Vitro

**DOI:** 10.1371/journal.pone.0064526

**Published:** 2013-05-28

**Authors:** Jing Sun, Guibo Sun, Xiangbao Meng, Hongwei Wang, Yun Luo, Meng Qin, Bo Ma, Min Wang, Dayong Cai, Peng Guo, Xiaobo Sun

**Affiliations:** 1 Key Laboratory of Bioactive Substances and Resources Utilization of Chinese Herbal Medicine, Ministry of Education, Institute of Medicinal Plant Development, Chinese Academy of Medical Sciences and Peking Union Medical College, Beijing, P. R. China; 2 Center for Translational Medicine and Jiangsu Key Laboratory of Molecular Medicine, Medical School of Nanjing University, Nanjing, Jiangsu, P. R. China; Virginia Commonwealth University Medical Center, United States of America

## Abstract

Doxorubicin (Dox) is an anthracycline antibiotic for cancer therapy with limited usage due to cardiotoxicity. Isorhamnetin is a nature antioxidant with obvious cardiac protective effect. The aim of this study is going to investigate the possible protective effect of isorhamnetin against Dox-induced cardiotoxicity and its underlying mechanisms. In an in vivo investigation, rats were intraperitoneally (i.p.) administered with Dox to duplicate the model of Dox-induced chronic cardiotoxicity. Daily pretreatment with isorhamnetin (5 mg/kg, i.p.) for 7 days was found to reduce Dox-induced myocardial damage significantly, including the decline of cardiac index, decrease in the release of serum cardiac enzymes and amelioration of heart vacuolation. In vitro studies on H9c2 cardiomyocytes, isorhamnetin was effective to reduce Dox-induced cell toxicity. A further mechanism study indicated that isorhamnetin pretreatment can counteract Dox-induced oxidative stress and suppress the activation of mitochondrion apoptotic pathway and mitogen-activated protein kinase pathway. Isorhamnetin also potentiated the anti-cancer activity of Dox in MCF-7, HepG2 and Hep2 cells. These findings indicated that isorhamnetin can be used as an adjuvant therapy for the long-term clinical use of Dox.

## Introduction

Doxorubicin (Dox), a member of the anthracycline family, continues to be one of the most effective anticancer drugs ever developed since its isolation in the early 1960s. This drug was proven to have an indispensable role in cancer chemotherapy. Dox is most widely used in the treatment of many types of hematologic and solid malignancies [1], [2]. Nevertheless, the clinical efficacy of Dox is seriously limited by cumulative, dose-dependent, and irreversible cardiotoxicity. Patients receiving Dox treatment can develop cardiomyopathy and subsequent severe congestive heart failure that is usually refractory to common medications, even many years after the cessation of Dox chemotherapy [3], [4]. Extensive studies have focused on identifying the methods or drugs capable of reducing this cardiotoxicity. To date, only dexrazoxane, which works as an antioxidant agent, is approved by the Food and Drug Administration to exhibit cardioprotective effect in Dox treatment. However, negative effects of this drug other than cardiac damage, such as hematological and hepatological toxicities were observed clinically [5]. Thus, a cardioprotective drug that can decrease heart damage without reducing the anticancer efficacy and without other negative effects during Dox therapy must be developed.

Despite intensive investigations on the Dox-induced cardiotoxicity, particularly, late onset cardiomyopathy has been continued for decades, the exact mechanisms of such toxicity are still difficult to elaborate. Most studies remain in favor of the view, among the proposed hypotheses, that the increased production of reactive oxygen species (ROS) and subsequent apoptosis are the pivotal adverse factors in the pathogenesis of Dox-induced cardiomyopathy [6]. The two main pathways that stimulating apoptosis are the intrinsic and the extrinsic pathways. A recent study reported that cardiomyocytes are consistently resistant to the extrinsic pathway [7]. The laboratory results of our previous study also proved that the intrinsic, not the extrinsic signaling pathway, is the main mechanism that participates in Dox-induced cardiomyocyte apoptosis [8]. The intrinsic apoptotic pathway is always initiated by the p53 tumor suppressor gene, a sensor of cellular stress, which is reportedly involved in Dox-induced cardiomyocyte apoptosis [9]. Recent reports indicated that the p53 tumor suppressor gene can directly regulate a host of Bcl-2 family proteins such as Bcl-2 anti-apoptotic and Bax pro-apoptotic proteins [10]. A study proposed that these proteins can regulate the collapse of the mitochondrial membrane potential, cytochrome c release, activation of caspase-9, and subsequent activation of caspase-3, which is the key executioner of apoptosis [11], [12].

Previous reports indicate that the mitogen-activated protein kinase (MAPK) family is responsible for the expression of p53 and Bcl-2 family-mediated apoptosis [13], [14], [15]. MAPK consists of three major signaling cascades: the extracellular signal-related kinases (ERK1/2), the c-Jun N-terminal kinases (JNK), and the p38 kinase (p38). Numerous studies suggest that MAPK activation is involved in cardiomyocyte apoptosis induced by Dox [10], [16].

**Figure 1:**
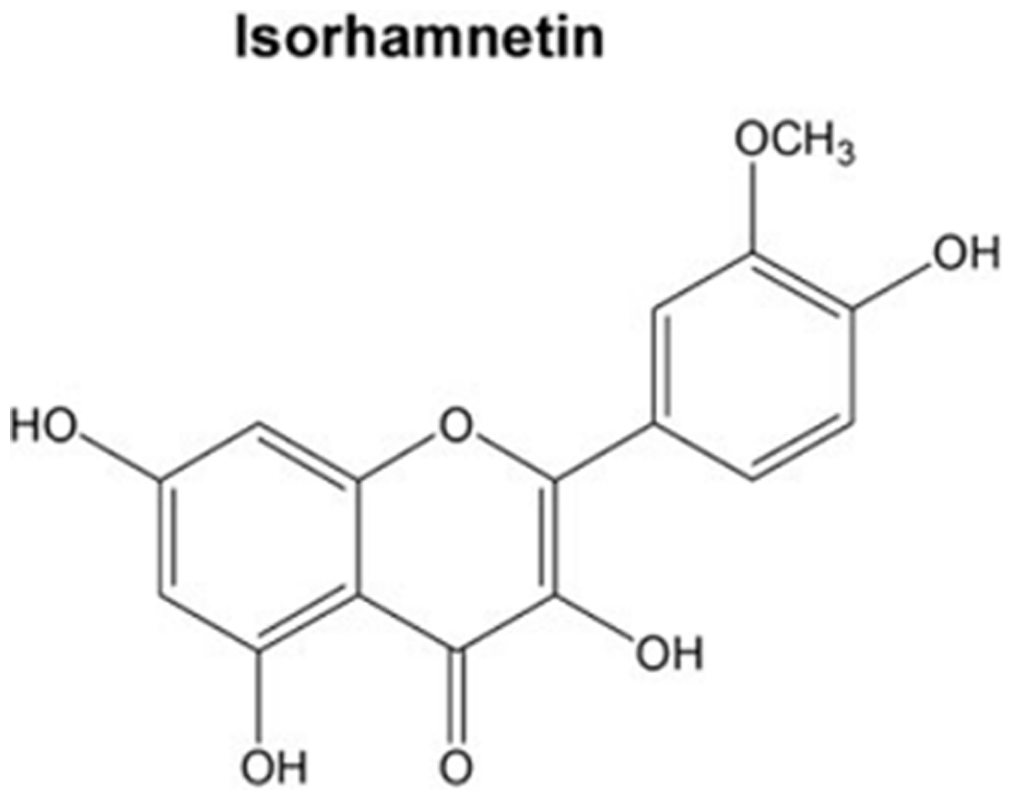
Molecular structure of isorhamnetin.

Isorhamnetin (Figure 1) is an abundant ﬂavonol aglycone in herbal medicinal plants, such as sea buckthorn (*Hippophae rhamnoides* L.) and *Ginkgo biloba* L., which are frequently used in the prevention and treatment of cardiovascular diseases [17], [18]. A number of studies suggested that isorhamnetin can protect endothelial cells from injury caused by oxidized low-density lipoprotein [19], decrease blood pressure [20], and alleviate the damages of ischemia-reperfusion (I/R) to ventricular myocytes [21]. Our laboratory study recently demonstrated that isorhamnetin can prevent H_2_O_2_-induced oxidative injury to H9c2 cardiomyocytes [22]. Isorhamnetin is also a powerful natural anti-cancer agent on many tumor cell lines [23], [24]. However, little is known about the possible protective effect of isorhamnetin against Dox-induced cardiotoxicity and the underlying mechanisms. Based upon the above facts, we investigated this possibility via in vivo and in vitro experiments. Moreover, we also elucidated the underlying mechanisms by investigating the involvement of mitochondria-dependent apoptotic pathway and MAPK signaling pathway.

## Materials and Methods

### Chemicals and Materials

Isorhamnetin (molecular weight = 316.27; purity >98%) was purchased from Shanghai Winherb Medical S&T Development (Shanghai, China). Dox was purchased from Shenzhen Main Luck Pharmaceuticals, Inc (Shenzhen, China). Cell culture materials were purchased from GIBCO (Grand Island, NY). The Cell Counting Kit-8 was obtained from Dojindo laboratory (Japan). Caspase-3 ﬂuorometric assay kits were acquired from BioVision (CA, USA). The fluorescent dye (JC-1) was purchased from Molecular Probes (Eugene, OR). The kits for determining malondialdehyde (MDA), glutathione peroxidase (GSH-Px), superoxide dismutase (SOD), and catalase (CAT) were purchased from Nanjing Jiancheng Institute of Biological Engineering (Nanjing, China). The kits for measuring lactate dehydrogenase (LDH), creatine kinase (CK) and aspartate aminotransferase (AST) were obtained from Biosino Bio-Technology and Science Incorporation (Hong Kong, China). Annexin V/propidium iodide (PI) apoptosis detection kit was obtained from Invitrogen (Eugene, USA). All antibodies were purchased from Santa Cruz Biotechnology (Santa Cruz, CA), whereas other chemicals were purchased from Sigma (St. Louis, MO).

### Animals and Experimental Protocols

Protocols were approved by the Animal Ethics Committee of Peking Union Medical College. Male Sprague-Dawley rats (Vital River Laboratories, Beijing, China), weighing approximately 300 g ±10 g (8 weeks of age), were used in this study. The animals were housed under standard laboratory conditions (temperature of 22°C ±1°C, humidity of 60%, and light from 6 a.m. to 6 p.m.) and allowed free access to standard rodent chow and water. The rats were randomly assigned to four groups.

1. Control group (Cont, n = 16): rats were intraperitoneally (i.p.) injected with saline;
2. Doxorubicin group (Dox, n = 16): rats were treated with Dox i.p. with a dose of 3 mg/kg every other day for a cumulative dose of 9 mg/kg, as previously described [25].
3. Isorhamnetin group (Iso, n = 16): rats were pretreated with isorhamnetin at a dose of 5 mg/kg i.p. every day for 6 days;
4. Isorhamnetin doxorubicin group (Dox+Iso, n = 16): rats were treated with isorhamnetin at a dose of 5 mg/kg i.p. prior to Dox for 7 days.

After 28 days, the rats were then sacrificed and the hearts were removed rapidly. The relative heart weight index (heart weight-to-body weight ratio) was determined. Blood was collected and centrifuged. The serum samples were assayed for LDH, CK, and AST activities. The left ventricles of the hearts were used for histopathological examination and other analyses.

### Measurement of Serum Enzyme Levels in Rats

After the rats were sacrificed, blood drawn from the inferior vena cava was allowed to clot. The levels of LDH, CK, and AST activities in the serum, as well as the pivotal diagnostic indicators of myocardial injury, were measured according to the manufacturer’s instructions (Nanjing Jiancheng Biotechnology Institute, China).

### Measurement of MDA Levels, and Antioxidant Enzyme Activities in Heart Homogenates

The MDA content and antioxidant enzymes, including SOD, CAT, and GSH-Px activities in heart homogenates were measured according to the manufacturer’s instructions.

### Heart Histopathological Examination

After fixation with 4% paraformaldehyde, the left ventricles of the hearts were trimmed and embedded in paraffin blocks, sectioned, stained with hematoxylin and eosin (HE), and examined under a light microscope (CKX41, 170 Olympus, Tokyo, Japan) by a pathologist blinded to the groups under study.

### Heart Immunohistochemical Examination

Anti-apoptotic effects were determined by immunohistochemistry with an antibody reacting to caspase-3. Briefly, heart sections were dewaxed, rehydrated through graded ethanol washes, and incubated with 3% hydrogen peroxide for 10 min. After antigen retrieval, the sections were obturated by incubation with normal goat serum for 60 min. The sections were continuously incubated with the mouse anti-caspase-3 monoclonal antibody at 4°C overnight, and then with the goat anti-mouse IgG secondary antibody for 1 h at room temperature. The sections were then stained with 0.5 g/L diaminobenzidine for 8 min, resulting in brown staining of the left ventricles of the hearts for viewing with an inverted microscope connected to a digital camera (CKX41, 170 Olympus, Tokyo, Japan).

### Cell Culture

H9c2 cardiomyocytes were obtained from the Cell Bank of the Chinese Academy of Sciences (Shanghai, China) and cultured in Dulbecco’s modified Eagle’s medium, supplemented with 10% (v/v) fetal bovine serum, 1% penicillin/streptomycin (v/v) and 2 mM L-glutamine. Human breast carcinoma cell line MCF-7, human hepatocellular carcinoma cell line HepG2 and laryngeal carcinomas cell line Hep2 cells were obtained from the Cell Bank of the Chinese Academy of Sciences (Shanghai, China) and cultured in RPMI 1640 medium with the supplements mentioned above. All the cells were maintained in a humidified incubator containing 5% CO_2_ at 37°C. The medium was changed every day, and the cells were in the exponential phase of growth before exposure to drugs in all experiments.

### Analysis of Cell Viability

The cell viability was analyzed using the Cell Counting Kit-8 (CCK8) assay. Prior to the treatment, H9c2 cardiomyocytes were plated at a density of 5×10^4^ cells/ml in 96-well plates and grown for 24 h. The cells were first treated for 6, 12, 24, and 36 h with different concentrations of Dox. Cell viability was determined according to the CCK8 assay kit user’s manual. Briefly, 10 µL of CCK8 solution was added to the culture medium, and then incubated for an additional 2 h. The optical density was measured at 450 nm wavelength, by using a microplate reader (MQX 200, BioTek Instruments, Winooski, VT, USA). The cells were pretreated with isorhamnetin for 12 h with final concentrations of 3.125, 6.25, 12.5, and 25 µg/ml, respectively, and then with 1 µM of Dox for an additional 36 h. The cell viability was evaluated as previously discussed.

Effects of isorhamnetin on the antitumor ability of Dox were tested in MCF-7, HepG2 and Hep2 cells. The cells were treated with Dox (1 µM) in presence or absence with isorhamnetin (6.25 µg/ml ) for 36 h. Cell viability was determined using CCK8 assay as mentioned above.

### Hoechst 33342 and Propidium Iodide (PI) Double Staining

In this study, Hoechst 33342 and PI double staining were used for qualitative analysis of the apoptotic cells. The H9c2 cardiomyocytes were cultured in 24-well plates for 24 h. After treatments, the cells were washed twice with phosphate-buffered saline (PBS), incubated with 10 µg/ml of Hoechst 33342 (Sigma) dye for 15 min at 37°C, and then added with 100 µg/ml of PI (Sigma). The stained nuclei were immediately observed by ﬂuorescence microscopy (DM4000B, Leica Wetzlar, Germany). Normal cells appeared to have intact nuclei with blue stain from Hoechst 33342. Early apoptotic cells with nuclear chromatin condensation and fragmentation were stained with bright blue by Hoechst 33342, whereas late apoptotic cells were stained with red/pink nuclei (Hoechst 33342 staining bright blue and PI staining red) [26].

### Terminal Deoxynucleotidyl Transferase-mediated dUTP Nick End Labeling (TUNEL) Staining

Apoptotic cardiac muscle cell in vivo and apoptotic H9c2 cardiomyocytes in vitro were visualized using the ApopTag Fluorescein In Situ Apoptosis Detection Kit according to the manufacturer’s instructions. The deparaffinized and rehydrated heart slices were incubated with proteinase K (20 µg/ml) for 15 min at room temperature. Rinsed with equilibration buffer, the slices were incubated with working-strength terminal deoxynucleotidyl transferase enzyme for 1 h at 37°C in a humidified chamber. After rinsing in a stop/wash buffer, the sections were incubated with working-strength anti-digoxigenin conjugate at room temperature for 30 min. The slices were stained with 4′6-diamidino-2-phenylindole and viewed under a fluorescence microscope (DM4000B, Leica Wetzlar, Germany). H9c2 cardiomyocytes were cultured on cover slips. After treatment with isorhamnetin and Dox, the cells were fixed with 4% neutral buffered formalin solution for 30 min. After two washes with PBS, the cells were treated in the same manner as what was previously described. The apoptotic cells were counted with at least 100 cells from four randomly selected fields in each treatment, and the values were expressed as percentages of the total number of cells.

### Assessment of Intracellular ROS Production

The level of intracellular ROS production was monitored using a total ROS detection kit. H9c2 cardiomyocytes were treated with 1 µM Dox, and the cells were harvested at 2, 4, 8, 12, 24, and 36 h. Intracellular ROS production was determined according to the manufacturer’s instructions. H9c2 cardiomyocytes were then pretreated with isorhamnetin for 12 h before exposure to Dox for 36 h. Fluorescence of the intracellular ROS production was detected on the microplate reader (Spectrafluor, TECAN, Sunrise, Austria) with an emission at 525 nm from 495 nm excitation or visualized using a fluorescence microscope (DM4000B, Leica Wetzlar, Germany).

### Assessment of LDH Release, MDA Levels, and Activities of SOD, CAT, and GSH-Px

H9c2 cardiomyocytes (1×10^5^ cells/well) were cultured in 6-well plates. The medium was collected for the measurement of the LDH release using an LDH assay kit according to the manufacturer’s instructions. The cells were harvested, ultrasonicated, and centrifuged at 1000 rpm for 5 min at 4°C. The supernate was used to assess the levels of MDA as well as the activities of SOD, CAT, and GSH-Px with corresponding detection kits.

### Determination of the Mitochondrial Membrane Potential (Δψ_m_)

5,5′,6,6′-Tetrachloro-1,1′,3,3′-tetraethylbenzimidazolyl-carbocya-nine iodide (JC-1) (Invitrogen) was used to determine the effect of isorhamnetin on the mitochondrial transmembrane potential. After the indicated treatments, H9c2 cardiomyocytes were harvested, loaded with 2 µM JC-1 in the dark at 37°C for 30 min following the manufacturer’s instructions, and then washed twice with PBS. Images of the cells labeled with JC-1 were observed under a fluorescence microscope (DM4000B, Leica Wetzlar, Germany).

### Analysis of Caspase-3 Activation

The activation of caspase-3 was determined using a fluorescein active caspase-3 staining kit (BioVision) according to the instructions supplied by the manufacturer. Briefly, H9c2 cardiomyocytes were collected after drug or vehicle treatments and then incubated on ice with 50 µL chilled lysate buffer for 10 min. After which, 50 µL of 2×reaction buffer (containing 10 mM dithiothreitol) and 5 µL of caspase-3 substrate (DEVD-AFC, 1 µM) were added to each sample. The specimens were then incubated for 2 h at 37°C. The fluorescence was detected in a microplate reader (SpectraFluor, TECAN, Sunrise, Austria) at 400 nm excitation wavelength and 505 nm emission wavelength.

### Protein Extraction and Western Blot Analysis

H9c2 cardiomyocytes treated with Dox or isorhamnetin were harvested, washed with PBS, and lysed with Cell Protein Extraction Reagent containing 1% phenylmethylsulfonyl fluoride on ice. The protein concentration was determined using a BCA kit (Pierce Corporation, Rockford, USA). Equal amounts of protein fractions were separated by electrophoresis on 10% sodium dodecyl sulfate polyacrylamide gels, in which the protein samples were evenly loaded. The proteins were then transferred onto nitrocellulose membranes (Millipore Corporation, USA) in Tris-glycine buffer at 110 V for 1 h. The membranes were blocked with 5% (w/v) non-fat milk powder in Tris-buffered saline containing 0.1% (v/v) Tween-20 (TBST) for 1 h, followed by incubation overnight with appropriate primary antibodies at 4°C. After washing with TBST thrice, the membranes were incubated with secondary antibodies for 2 h at room temperature. The membranes were washed again with TBST three times, and the blots were developed using an enhanced chemiluminescence (ECL) solution. The results were visualized by ECL with the Image Lab Software (Bio-Rad, USA). The left ventricles of the rat hearts were homogenated and Western blot analysis was performed in the same manner described above.

### Flow Cytometric Detection of Apoptosis

The percentage of early apoptosis and necrosis was detected using Annexin V-FITC/PI apoptosis kit for ﬂow cytometry in MCF-7, HepG2 and Hep2 cells. After treatments as mentioned above, the cells were harvested and washed twice with cold PBS, and then incubated with 5 µL FITC-Annexin V and 1 µL PI working solution (100 µg/mL) in the dark at room temperature for 15 min. Cellular ﬂuorescence was measured by ﬂow cytometry analysis with a FACS Calibur Flow Cytometerﬂow cytometer (BD Biosciences, USA).

### Statistical Analyses

The results are expressed as means ± standard deviation. All statistical analyses were performed using the Student’s t-test or ANOVA with the Prism 5.00 software. Statistical significance was considered at P<0.05.

### Ethics Statement

This study was performed following the regulations of the Chinese Guide for the Care and Use of Laboratory Animals published by the United States National Institutes of Health Publication No. 85-23, revised (1996).

## Results

### Isorhamnetin Protected against Dox-induced Cardiotoxicity in Rats

**Figure 2:**
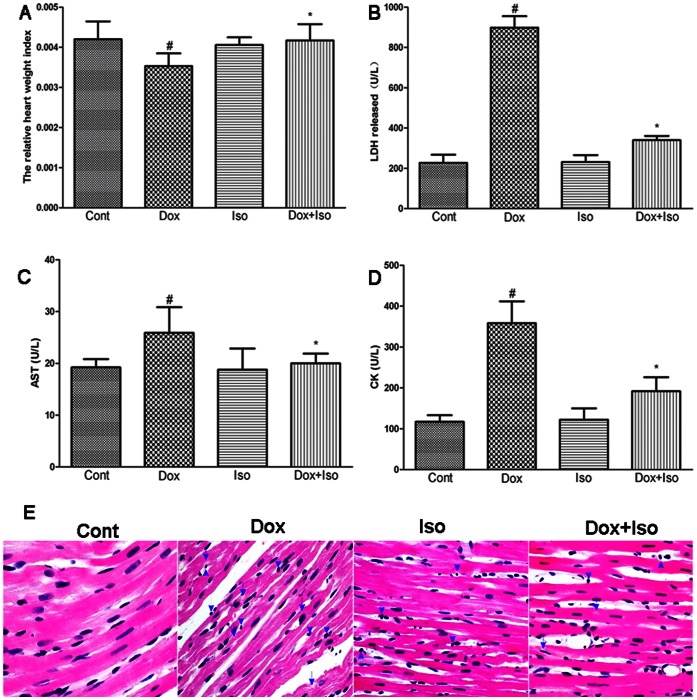
Effects of isorhamnetin on Dox-induced myocardial injury in vivo. Rats were intraperitoneally (i.p.) treated with vehicle or Dox (3 mg/kg every other day for a cumulative dose of 9 mg/kg) with isorhamnetin pretreatment (5 mg/kg i.p. before Dox administration). After 28 days, the relative heart weight index (heart weight–body weight ratio, g/g) was measured (A). The effects of isorhamnetin treatment on LDH release (B), AST (C), and CK (D) levels were detected according to the manufacturer’s instructions. The effects of isorhamnetin on histological changes in the rat hearts was measured by HE staining (E); Cont, vehicle treatment; Iso, isorhamnetin treatment; Dox, doxorubicin treatment; Dox+Iso, isorhamnetin and doxorubicin co-treatment. Dotted arrow indicated infiltrated neutrophilic granulocyte, and solid arrow did cytoplasmic vacuolation. Results are represented as means ± SD (n = 9 per group). ^#^P<0.05 vs. Cont; *P<0.05 vs. Dox-treated group.

We first evaluated the general toxicity of Dox and isorhamnetin to examine whether isorhamnetin was able to protect Dox-induced cardiac injury in rats. At the end of the treatment period, 16 rats in the control and isorhamnetin groups remained alive and healthy. Meanwhile, 31.25% (5 out of 16) and 6.25% (1 out of 16) rats died in the Dox and Dox+Iso groups, respectively, and were consequently excluded from the analysis. At a later period, all surviving rats in the Dox group appeared weak, with scruffy fur and red exudate around the eyes and noses. In addition, abdominal distension was observed in 2 of 11 Dox-treated rats alive but none from other groups. The administration of Dox also led to a significant decrease (P<0.05) in heart-to-body weight ratio compared with that of the control group. In contrast, the pretreatment with isorhamnetin (5 mg/kg before Dox administration) in rats resulted in an evident recovery of heart weight/body weight (Figure 2A).

The levels of the cardiac enzymes (LDH, AST, and CK) were shown in Figures 2B, 2C, and 2D. Pretreatment of isorhamnetin significantly reduced the Dox-induced increase of cardiac enzymes in serum, and the group with isorhamnetin alone showed no evident abnormalities from the control.

The morphological changes in the myocardial damage were also detected by HE staining. Compared with the control group, the group with Dox treatment showed serious disorganization of myofibrillar arrays, cytoplasmic vacuolization, and intense infiltration with neutrophil granulocytes. Isorhamnetin pretreatment markedly reduced the pathological changes, and no evident abnormalities were observed in the group with isorhamnetin alone (Figure 2E).

### Isorhamnetin Protected against Dox Induced Cytotoxicity in Rat H9c2 Cardiomyocytes

**Figure 3:**
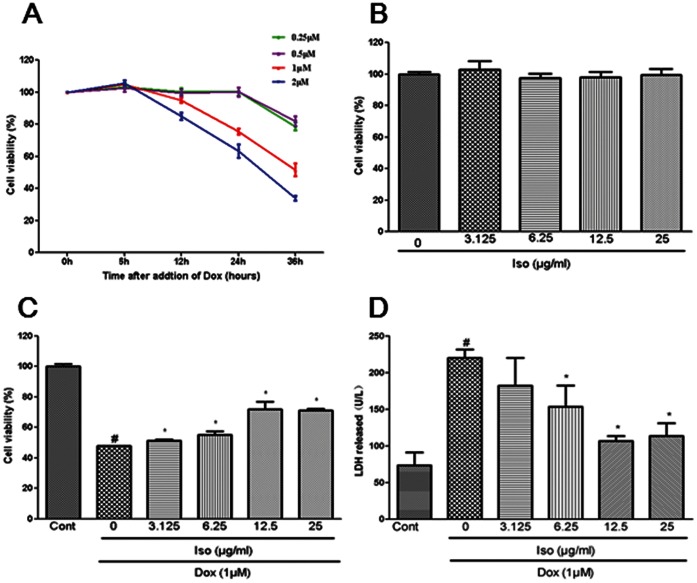
Effects of Dox and isorhamnetin on cell viability and LDH release in vitro. H9c2 cardiomyocytes were treated for 6, 12, 24 and 36 h with different concentrations of Dox, and cell viability was determined by CCK8 assay (A). H9c2 cardiomyocytes were treated with different concentrations of isorhamnetin for 12 h, and cell viability was expressed as the relative percentage of control group (B). Control and isorhamnetin treated cells were further exposed to 1 µM Dox for 36 h; the cell viability (C) and LDH release (D) were measured. Results are represented as means ± SD from three independent experiments. ^#^P<0.05 vs. Cont; *P<0.05 vs. Dox-treated group.

The effect of Dox on cell viability was first determined using CCK8 assay to evaluate the capacity of isorhamnetin to protect against Dox-induced cardiac injury in vitro. H9c2 cardiomyocytes were treated with different Dox concentrations (0.25, 0.5, 1, and 2 µM) for 6, 12, 24, and 36 h, respectively. The results showed that Dox induced a dose- and time- dependent toxicity in H9c2 cardiomyocytes as indicated by the decreased cell viability (Figure 3A). Dox (1 µM) treatment provoked about 50% of cell death at 36 h, therefore, 1 µM of Dox for 36 h was the condition chosen for the following experiments.

The direct effect of isorhamnetin on H9c2 cardiomyocytes was analyzed before testing the role of isorhamnetin-mediated protection against Dox-induced cell damage. Cells were treated for 12 h with isorhamnetin with varying concentrations ranging from 3.125 µg/ml to 25 µg/ml. As indicated in Figure 3B, the difference in cell viability between isorhamnetin-treated and control groups was not observed, demonstrating that none of the tested isorhamnetin concentrations can induce cell injury in H9c2 cardiomyocytes.

H9c2 cardiomyocytes pretreated with isorhamnetin (3.125 µg/ml to 25 µg/ml) for 12 h were further exposed to 1 µM of Dox for 36 h. Viability and LDH release were evaluated to assess whether treatment with isorhamnetin repressed the Dox-induced cytotoxic effect. As shown in Figures 3C and 3D, pretreatment with 3.125, 6.25, and 12.5 µg/ml isorhamnetin increased cell viability and decreased the LDH leakage in a dose-dependent manner. Higher isorhamnetin concentrations (up to 25 µg/ml) did not improve cell viability and LDH leakage, therefore, all subsequent experiments were performed with 12.5 µg/ml of isorhamnetin.

### Isorhamnetin Inhibited Intracellular ROS Production and Enhanced Antioxidant Capacity in H9c2 Cardiomyocytes and Cardiac Tissues

**Figure 4:**
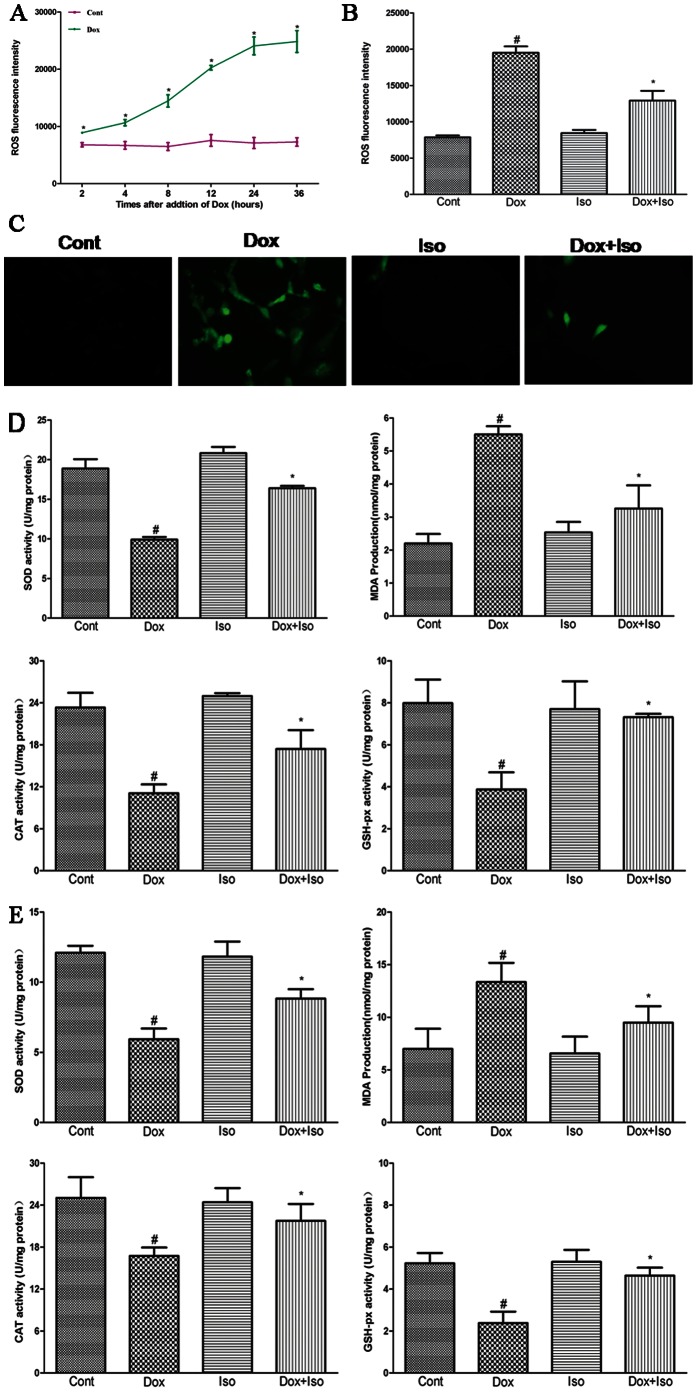
Effects of Dox and isorhamnetin on cell viability and LDH release in vitro.Effects of Dox and isorhamnetin on antioxidant capacity in vivo and in vitro. H9c2 cardiomyocytes were treated with vehicle or Dox for 0 h to 36 h and ROS ﬂuorescence was detected on the automatic microplate reader at different times (A). The effects of isorhamnetin on the ROS production were also measured with the automatic microplate reader and visualized by fluorescence microscopy (B and C). Effects of isorhamnetin on activities of SOD, MDA, CAT, and GSH-Px in H9c2 cardiomyocytes (D), and rat heart tissues (E) were measured by corresponding detection kits. Cont, vehicle treatment; Iso, isorhamnetin treatment; Dox, doxorubicin treatment; Dox+Iso, isorhamnetin and doxorubicin co-treatment. Results are represented as means ± SD (n = 3–9 per group). ^#^P<0.05 vs. Cont; *P<0.05 vs. Dox-treated group.

A body of evidence suggests that excess production of ROS is the primary mechanism involved in Dox-induced cardiotoxicity [9]. Thus, we examined the generation of intracellular ROS and the activity of associated antioxidant enzymes. As shown in Figure 4A, Dox treatment significantly increased ROS production with a concentration that induced apoptosis (1 µM) as early as 2 h after treatment, with a further increase of 4 h to 36 h, whereas pretreatment with isorhamnetin significantly decreased the generation of ROS (Figures 4B and 4C). These results suggested that isorhamnetin can inhibit Dox-induced intracellular ROS generation.

Subsequently, the effects of Dox and isorhamnetin on antioxidant enzyme activities were further evaluated in vivo and in vitro. As shown in Figures 4D and 4E, Dox caused oxidative stress damage both in rat heart and H9c2 cardiomyocytes as indicated by decreases in SOD, CAT, and GSH-Px activities and increase in lipid peroxidation (MDA) production, whereas these changes were effectively improved by isorhamnetin.

### Isorhamnetin Ameliorated Dox-induced Apoptotic Damage in vivo and in vitro

**Figure 5:**
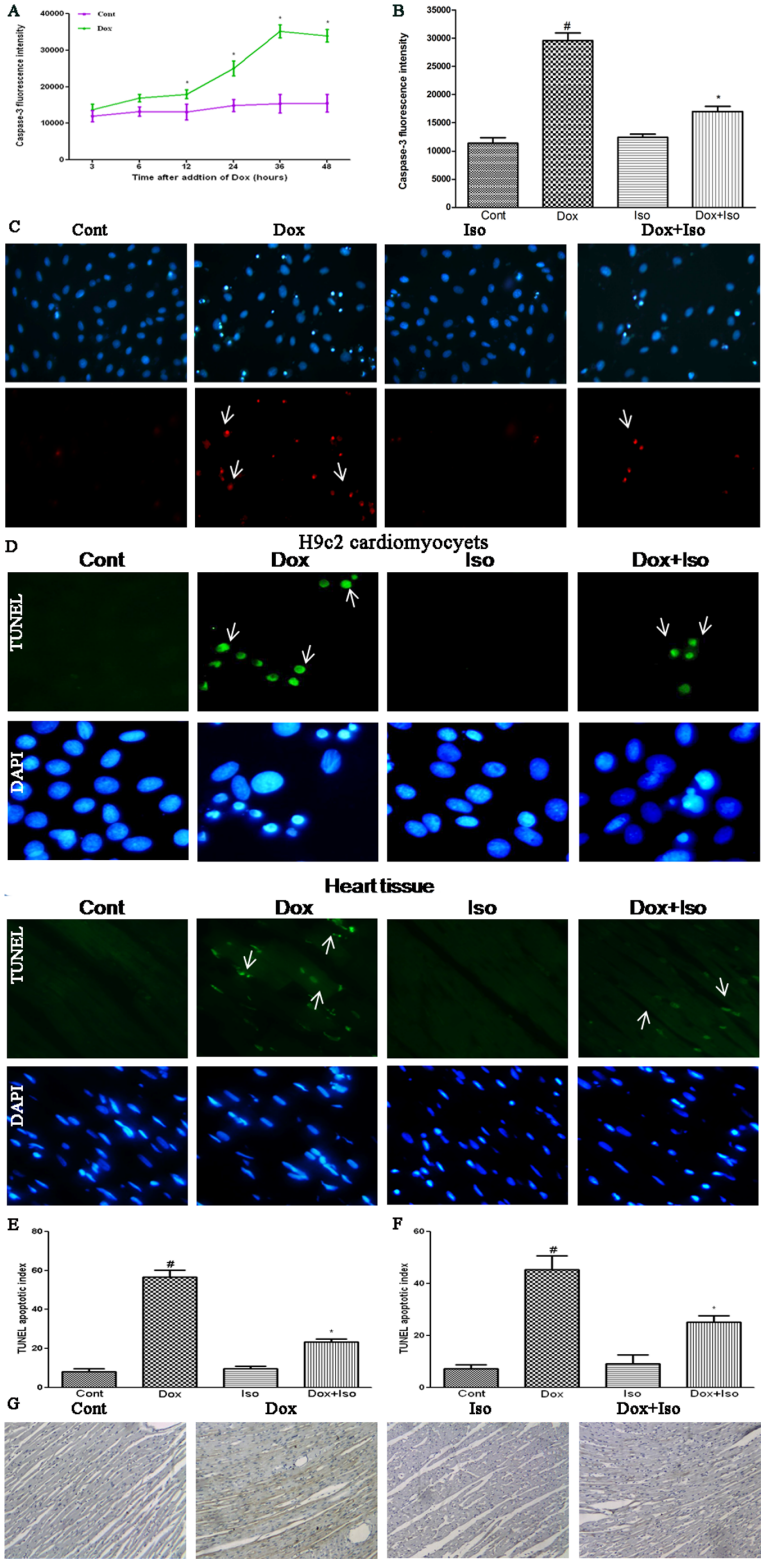
Effects of Dox and isorhamnetin on apoptosis in vivo and in vitro. H9c2 cardiomyocytes were incubated during increasing times with or without Dox (1 µM) and caspase-3 activity (units per microgram of protein) was assayed as described in Materials and Methods (A). Control and isorhamnetin-treated cells were further exposed to 1 µM of Dox for 36 h; caspase-3 fluorescence and Hoechst 33342 and propidium iodide (PI) double staining were used for the qualitative and quantitative analyses of the apoptotic cells (B and C). The internucleosomal DNA fragmentation was also determined by TUNEL assay in vitro and in vivo (D, scale bar). The TUNEL apoptotic index was determined by calculating the ratio of TUNEL-positive cells to total cells (E and F). Rat heart sections were also analyzed by immunohistochemistry with an antibody reactive to caspase-3 (G). Cont, vehicle treatment; Iso, isorhamnetin treatment; Dox, doxorubicin treatment; Dox+Iso, isorhamnetin and doxorubicin co-treatment. Results are represented as means ± SD (n = 3–9 per group). ^#^P<0.05 vs. Cont; *P<0.05 vs. Dox-treated cells.

Caspase-3 activity, a biomarker of apoptosis, was detected. H9c2 cardiomyocytes were briefly exposed to 1 µM of Dox at varying durations to assess whether this cytotoxic effect was associated with apoptosis. As shown in Figure 5A, the activation of caspase-3 induced by Dox started at 12 h of incubation and reached a peak at 36 h. These results indicated that Dox was able to activate caspase-3 and induce apoptosis in H9c2 cardiomyocytes.

To confirm whether the protective function of isorhamnetin was connected with apoptosis, fluorescence detection of caspase-3 and H33342/PI staining were used in H9c2 cardiomyocytes. As shown in Figures 5B and 5C, pretreatment of isorhamnetin with the concentration of 12.5 µg/ml for 12 h exhibited a strong anti-apoptosis effect.

TUNEL staining was used in both H9c2 cardiomyocytes and rat myocardial tissues. As revealed in Figure 5D, Dox-treated H9c2 cardiomyocytes resulted in significant internucleosomal DNA fragmentation. By contrast, pretreatment with isorhamnetin effectively ameliorated Dox-induced DNA fragmentation. The percentage of TUNEL-positive cells was also calculated. As presented in the histogram (Figure 5E), TUNEL-positive rate of cells treated with Dox increased to 62.70%, whereas those pretreated with isorhamnetin decreased to 22.17%. In accordance with the in vitro results, DNA fragmentation was also evident in the rat heart tissue injected i.p. with Dox and evidently improved with isorhamnetin pretreatment (Figures 5D and 5F). The protein expression of caspase-3 in heart tissues was also assessed by immunohistochemistry. Isorhamnetin pretreatment exhibited a significant protective effect (Figure 5G). However, treatment with isorhamnetin alone exhibited no significant effect on the apoptotic events in both in vivo and in vitro experiments. These results indicated that isoharmnetin can protect against Dox-induced apoptosis in cardiomyocytes.

### Anti-apoptotic Effects of Isorhamnetin against Dox are Related to Mitochondria-dependent Apoptotic Pathway

**Figure 6:**
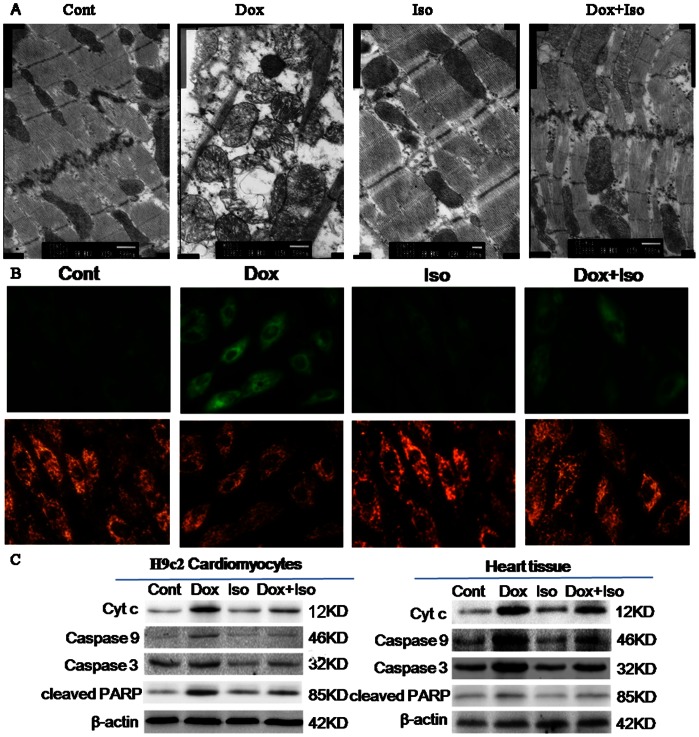
Effects of Dox and isorhamnetin on mitochondrial apoptotic pathway in vivo and in vitro. Mitochondrial injuries in rat heart tissues were observed by transmission electron microscopy (A). H9c2 cardiomyocytes stained with JC-1 dye were visualized by fluorescence microscopy (B). Cytochrome c (cyt c), caspase-9/3, and PARP in rat heart tissues and H9c2 cardiomyocytes were determined by western blot analysis (C). Cont, vehicle treatment; Iso, isorhamnetin treatment; Dox, doxorubicin treatment; Dox+Iso, isorhamnetin and doxorubicin co-treatment.

The mitochondria-dependent apoptotic pathway was considered the main mechanism underlying Dox-induced cardiomyocyte apoptosis. From the overall view provided by the transmission electron microscopy, heart tissue in 28-day Dox-treated rats appeared clear abnormalities, including cytoplasmic vacuolization, mitochondrial edema, myocardial fiber loss, chromatin condensation, and myocyte necrosis. These structural damages were partly alleviated by pretreatment with isorhamnetin (Figure 6A). JC-1 measures were also employed to assess Δψm in H9c2 cardiomyocytes. Green ﬂuorescence emission of monomeric JC-1 in the cytosol and red ﬂuorescence emission of aggregate JC-1 in the mitochondria were visualized under a fluorescence microscope. The results also revealed that Dox-induced Δψm depolarization was mitigated by isorhamnetin pretreatment to a certain extent (Figure 6B). These results demonstrated that isorhamnetin can reverse mitochondrial dysfunction both in vivo and in vitro. Further studies indicated that isorhamnetin can prevent Dox-induced release of cytochrome c, activation of caspase-9/−3, and cleavage of poly (ADP-ribose) polymerase (PARP) by immunoblotting assay (Figure 6C).

### Isorhamnetin Modulated the Expression of Bcl-2 Family Proteins and p53 both in vivo and in vitro

**Figure 7:**
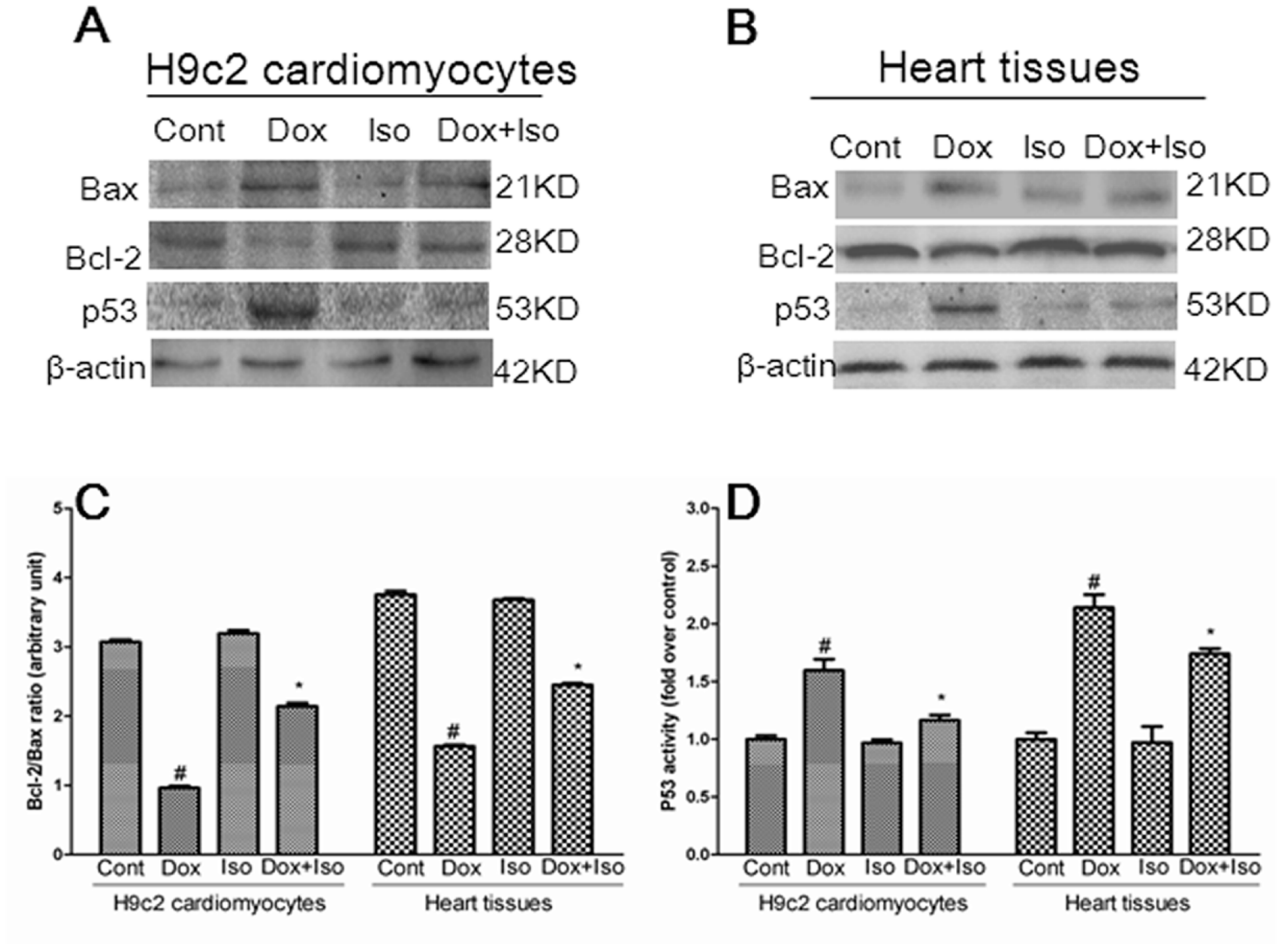
Effects of Dox and isorhamnetin on expression of Bcl-2 family proteins in vivo and in vitro. The expression levels of Bcl-2, Bax, and P53 were detected using an immunoblotting assay in vitro (A) and in vivo (B) and expressed as the fold changes over the control (C and D). Cont, vehicle treatment; Iso, isorhamnetin treatment; Dox, doxorubicin treatment; Dox+Iso, isorhamnetin and doxorubicin co-treatment. Results are represented as means ± SD from three independent experiments. ^#^P<0.05 vs. Cont; *P<0.05 vs. Dox-treated group.

The effects of isorhamnetin on the expression levels of Bax, Bcl-2, and p53 proteins in heart tissues and H9c2 cardiomyocytes were further evaluated using western blot analysis. As shown in Figures 7A and 7B, Dox-induced apoptosis was accompanied by a significant upregulation of the Bax expression and downregulation of the Bcl-2 expression. However, pretreatment with isorhamnetin blocked these effects. Statistical calculation of the Bcl-2/Bax expression ratio revealed that the isorhamnetin can modulate the disturbances of Bcl-2 and Bax expression induced by Dox (Figure 7C). The protective effect of isorhamnetin against Dox-induced cardiotoxicity was also related with the amelioration of p53 expression, as shown in Figures 7A and 7D.

### MAPK Signaling Pathway was Involved in the Anti-apoptotic Effect of Isorhamnetin

**Figure 8:**
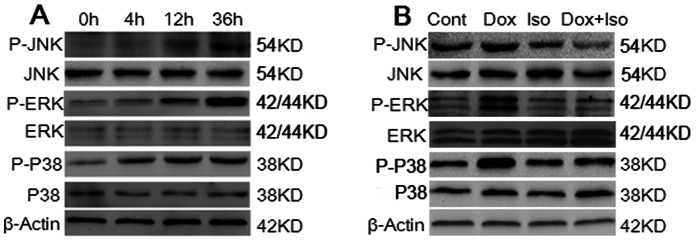
Effects of Dox and isorhamnetin on activiation of MAPK in vitro. H9c2 cardiomyocytes exposed to 1 µM of Dox for the indicated times (0 h to 36 h) were subjected to western blot analysis (A). H9c2 cardiomyocytes were treated with vehicle or Dox (1 µM, 36 h) with or without isorhamnetin (12.5 µg/ml) for 12 h prior to Dox exposure. The expression levels of ERK1/2, p38, and JNK were detected by immunoblotting assay. Cont, vehicle treatment; Iso, isorhamnetin treatment; Dox, doxorubicin treatment; Dox+Iso, doxorubicin and isorhamnetin co-treatment.

Accumulating evidence indicates that MAPK play a major role in apoptosis signaling [1]. Thus, we investigated the effects of Dox and isorhamnetin on the expression of the MAPK signaling pathway, including total and phosphorylated (active form) JNK, ERK, and p38 MAPK, by western blot analysis with varying durations (4 h to 36 h). Figure 8A indicated that the expression levels of p-JNK, p-ERK, and p-p38 were markedly increased with the treatment of 1 µM of Dox. Phosphorylation of ERK, JNK, and p38 were clearly visiable at 4 h after Dox treatment and remained active until 36 h. No changes were observed in total ERK, JNK, and p38 protein levels in the presence of Dox. These results indicated that MAPK signaling pathway was involved in Dox-induced apoptosis of H9c2 cardiomyocytes. Therefore, 36 h was chosen for the optimized time point for the following experiments. As shown in Figure 8B, pretreatment with isorhamnetin significantly inhibited the expression of p-ERK1/2, p-JNK, and p-p38 induced by Dox, suggesting that the MAPK signaling pathway was involved in the anti-apoptotic effect of isorhamnetin.

### Isorhamnetin Potentiated the Anticancer Activity of Dox in MCF-7, HepG2, and Hep2 Cells

**Figure 9:**
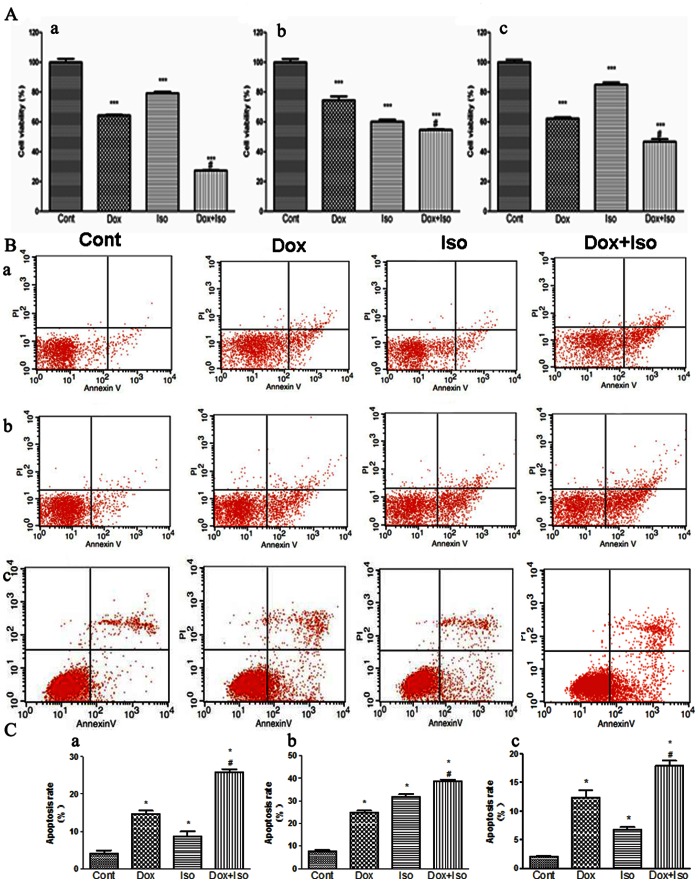
Effects of isorhamnetin on the antitumor ability of Dox in vitro. A series of cancer cell lines, including MCF-7 (a), HepG2 (b), and Hep2 (c) cells were treated with Dox (1 µM ) and isorhamnetin (6.25 µg/ml) alone or co-incubation for 36 h. The cell viability was measured by CCK8 assay (A). The cells stained with Annexin V-FITC/PI measured by Flow cytometry (B) and statistical analysis of the ﬂow cytometry was shown (C). Cont, vehicle treatment; Iso, isorhamnetin treatment; Dox, doxorubicin treatment; Dox+Iso, doxorubicin and isorhamnetin co-treatment. Results are represented as means ± SD from three independent experiments. *P<0.05 vs. Cont; #P<0.05 vs. Dox-treated group.

To determine whether isorhamnetin affected the antitumor ability of Dox, a series of cancer cell lines, including MCF-7, HepG2 and Hep2 cells were used in the experiments. As shown in Figure 9A, Dox co-treated with isorhamnetin produced the maximum decrease in cancer cell ability compared with either drug used alone. Apoptosis in MCF-7, HepG2 and Hep2 cells was further evaluated through the quantitative analysis of FITC-Annexin V/PI staining by flow cytometry. As shown in Figures 9B and 9C, the percentage of apoptotic cells increased up to 25.71%, 38.61%, 17.92% in MCF-7, HepG2 and Hep2 cells respectively in co-treatment groups, higher than that in either drug used alone groups. These findings confirmed that isorhamnetin can potentiate the antitumor ability of Dox.

## Discussion

The clinical efficacy of Dox, an antibiotic antitumor drug, is severely limited by its dose-dependent cardiotoxicity, which leads to irreversible degenerative cardiomyopathy and heart failure in cancer patients [27]. This study revealed that isorhamnetin can protect against Dox-induced chronic cardiotoxicity and potentiate its anticancer activity. To the best of our knowledge, this is the first report which proved that isorhamnetin might be a potential candidate in preventing heart damage in Dox-treated cancer patients. Isorhamnetin exists in large quantities in sea buckthorn, Ginkgo, and other plant sources which are frequently used in the prevention and treatment of cardiovascular diseases. Isorhamnetin reportedly exhibits numerous biological activities such as scavenging of free radicals, increasing the resistance of low-density lipoprotein to oxidation, protecting I/R induced ventricular cell injury, and antitumor activity [28], [29].

In the present study, a chronic cardiac injury model was set up, in this model, rats were injected i.p. with isorhamnetin in a cumulative dose of 9 mg/kg, which was just above the clinical cardiac toxicity critical value induced by Dox [25]. Compared with the experimental model of Dox-induced injury of rat myocardium, injection with isorhamnetin reduced the mortality and the cardiac index, decreased the level of serum cardiac enzymes, and improved the histological damage in the heart, suggesting that isorhamnetin exerts a prominent protective action against Dox-induced myocardial injury in vivo. In accordance with this result, in vitro experiments of the present study also showed that pretreatment with isorhamnetin can effectively increase the cell viability and inhibit the release of LDH induced by Dox in H9c2 cardiomyocytes, which are considered as the biomarker of cardiotoxicity [30].

Most studies favor the free radical-induced oxidative stress that plays a pivotal role in Dox-induced cardiotoxicity [31]. Excessive production of ROS is identified as a contributor to the damage of nuclear DNA, changes in calcium handling, restrain from cell survival and protein synthesis, disruption of sarcomere stability, and eventual cardiomyocyte death [32]. Due to their phenolic hydroxy structure, flavonoid compounds can act as strong natural antioxidants via combining directly with ROS and activating the antioxidant defense systems [33]. The present study was primarily designed to examine the potent protective effect of isorhamnetin on Dox-induced oxidative stress. The results demonstrated that pretreatment with isorhamnetin inhibited cellular ROS production and up-regulated the levels of CAT, GSH-Px, and SOD, as well as reduced MDA generation in rats and H9c2 cardiomyocytes.

Previous studies demonstrated that the apoptotic death of cardiomyocytes is the most direct cause of Dox-induced cardiotoxicity [34]. Apoptosis depends on the activation of caspases. Caspases are a family of aspartate-specific cysteine proteases that play key roles in regulating the cellular and biochemical changes associated with apoptosis. Caspase-3 is the key executioner of apoptosis and is reportedly activated by Dox [35]. In the present study, treatment with 1 µM of Dox was shown to initiate the activation of caspase-3 at 12 h and reach a peak at 36 h in H9c2 cardiomyocytes. The protective effect of isorhamnetin against apoptosis damage was confirmed by detection of caspase-3 fluorescence, examination of heart immunohistochemistry, Hoechst 33342/PI staining, and measurement of DNA fragmentation by TUNEL staining.

Two distinct pathways stimulate caspases: the intrinsic (mitochondrial-dependent) apoptotic pathway and the extrinsic (death receptor-dependent) pathway [36]. Our laboratory has previously reported that the mitochondrial-dependent apoptotic pathway was the dominant mechanism involved in Dox-induced myocardial apoptosis [8]. To confirm whether the mechanism underlying the anti-apoptotic effect of isorhamnetin was related to the mitochondrial-dependent apoptotic pathway, we detected a series of changes involved in mitochondrial injury. In apoptotic cells, the mitochondrial membrane potential (Δψm) is impaired, causing the opening of permeability transition pores. This occurrence induces the release of cytochrome c from mitochondria into cytoplasm and the activation of caspase-9/−3 as well as the subsequent cleavage PARP [37]. Our results demonstrated that isorhamnetin can markedly prevent Δψm dissipation, cytochrome c release, caspase-9/−3 activation, and PARP cleavage induced by Dox, both in vivo and in vitro. The activation of mitochondrial-dependent apoptotic pathway has been proposed to depend on the induction of the p53 tumor-suppressor protein, which is also associated with Dox-induced cardiomyocytes apoptosis [38]. p53 is a transcription factor that plays a critical role in the cellular response to DNA damage and can directly regulate the gene products of Bcl-2 family proteins, including the Bax pro-apoptotic protein and Bcl-2 anti-apoptotic protein, which subsequently interfere with cytochrome c release and ultimately initiate apoptosis [39]. Our results revealed that p53 was maintained at a low level in the control group cells. Dox induced an increase in the expression of p53, but pretreatment with isorhamnetin counteracted the effect. The Dox treatment also induced an increase in Bax expression and a decrease in Bcl-2 protein expression compared with the control group. Isorhamnetin can up-regulate Bcl-2, and down-regulate Bax, both in vitro and in vivo.

Accumulating evidence indicate that the MAPK signaling pathways are the key intermediates in the induction of p53 and Bcl-2 family-mediated apoptosis [40]. The changes in MAPK were examined extensively in Dox-induced cardiac toxicity; however, the information reported was varied. Liu et al. reported that the phosphorylation of ERK1/2 increased persistently, whereas p-JNK and p-p38 remained unchanged in H9c2 cardiomyocytes treated with Dox [10]. By contrast, in another report, p38 MAPK was shown to play a crucial role in the regulation of cell apoptosis in both in vitro and in vivo gene knockout studies [41]. In this regard, we suspect that the MAPK is differentially regulated, at least in part, depending on the doses of Dox and the experimental conditions used. Hence in this study, a time course of changes in MAPK in H9c2 cardiomyocytes, with administration of 1 µM of Dox, was conducted. Our data showed that the expression of p-JNK, p-ERK, and p-p38 were evident after 4 h of treatment and persistently active until 36 h, suggesting that these kinases play a dominant role in the progression of myocardial damage induced by Dox. Subsequent experiments revealed that pretreatment of isorhamnetin can neutralize the Dox-induced phosphorylation of JNK, ERK1/2, and p38.

ERK1/2 pathway is well known to play a key role in some cellular processes such as proliferation, survival, transformation, and apoptosis [42]. It is extensively described that ERK phosphorylation is protective in the heart and other cells [43], [44]. But more recently, a large body of evidence has accumulated to show that ERK1/2 may mediate both destructive and protective effects in different systems. The roles played by ERK1/2 tend to be inﬂuenced by the cell type, drug concentration and duration of exposure, and on the type of assay used to monitor apoptosis or cell survival [45]. A recent study has revealed that a sustained and delayed ERK1/2 phosphorylation induced by Dox is responsible for inducing p53 activation and mitochondrial apoptosis in H9c2 cardiomyocytes [10]. In addition, a growing body of research supports the view that sustained and/or delayed activation of ERK1/2 contributes to the occurrence of apoptosis [46], [47]. Our present results showed that p-ERK1/2 were persistently actived by Dox and pretreatment of isorhamnetin reduced that phosphorylation, indicating that the cardioprotective effect of isorhamnetin was associated with inhibition of the sustained ERK1/2 phosphorylation induced by Dox.

Another issue discussed in the present study was the effect of isorhamnetin on the antitumor activity of Dox. Evidence suggests that isorhamnetin is a powerful natural anticancer agent [23], [24]. We question whether isorhamnetin treatment will alter the anti-cancer function of Dox. Our in vitro studies were performed on a series of cancer cell lines (i.e., MCF-7, HepG2, and Hep2 cells). Our results showed that the combination of isorhamnetin and Dox induced the highest decrease in cell viability and increase in apoptosis of MCF-7, HepG2, and Hep2 cancer cells compared with either drug used alone. These results indicated that isorhamnetin potentiated the anticancer effect of Dox. The possible interpretation for the different effects of isorhamnetin on H9c2 cardiomyocytes and tumor cells, is that the effect occurs in a cell-dependent manner. Accordingly, this finding of cell-selective characteristic was also observed in other reports [48].

### Conclusion

In summary, this study demonstrated that isorhamnetin elicits a typical cardioprotective effect on Dox-related cardiotoxicity in vivo and in vitro. This protective effect was correlated to the inhibition of oxidative stress and subsequent mitochondrial-dependent apoptotic pathway and MAPK signaling pathway. Thus, isorhamnetin can be a novel candidate for the combination with Dox to protect against Dox-induced cardiotoxicity.
